# Supplementary material for: Bioinformatics and Screening of a Circular RNA-microRNA-mRNA Regulatory Network Induced by Coxsackievirus Group B5 in Human Rhabdomyosarcoma Cells
Source: Int J Mol Sci. 2022 Apr 22;23(9):4628. doi: 10.3390/ijms23094628 (PMC9101002; doi:10.3390/ijms23094628)
Supplement: Supplementary file 1 [file ijms-23-04628-s001.zip › Table S6 Primers used for analysis.pdf]

Table S6 Primers used in this study

A Primers used for RT-qPCR analysis		
**circRNA**	**Forward primer 5'–3'**	**Reverse primer 5'–3'**
ACTB	GCGTGACATTAAGGAGAAGC	CCACGTCACACTTCATGATGG
novel_circ_0010492	CTTTGGAGCCTGGGAGACTC	CGTGTTCTGCTCTGTTGGTC
hsa_circ_0075346	TGAAGATCCTTGACTTTGGCCT	GACTGTCACATTTACTGTCGCTCAT
novel_circ_0001066	TGTGAAATATCTCAGCAACAGTGG	ATCAGCTCCGTAAATAGCACCAT
hsa_circ_0008378	GACCCAGCCGACCAATTAATAT	ATGGAACTACTGATTTGCTGCAC
hsa_circ_0004390	AGCCGTCTTCTGTCATGAAC	GCTCCAATTCTGTCATTCCACG
novel_circ_0002006	TTGTACTCCAACGCCACCCT	TCCACTCGTCTGGTCCGCAT

B Primers used for miRNA analysis			
**miRNA**	**Reverse Transcription primer 5'–3'**	**Forward primer 5'–3'**	**Reverse primer 5'–3'**
miR-4526	GTCGTATCGACTG-CAGGGTCCGAGGTATTCG-CAGTCGATACGACAGCGGC	GCGCTGACGCGGGCTG	ACTG-CAGGGTCCGAGGTATT
miR-3123	GTCGTATCGACTG-CAGGGTCCGAGGTATTCG-CAGTCGATACGACGATTAA	GGCCGGCCAGAGAATTGT	ACTG-CAGGGTCCGAGGTATT
miR-152-3p	GTCGTATCGACTG-CAGGGTCCGAGGTATTCG-CAGTCGATACGACCCAAGT	CGGCTCAGTGCATGACAGA	ACTG-CAGGGTCCGAGGTATT
miR-29b-3p	GTCGTATCGACTG-CAGGGTCCGAGGTATTCG-CAGTCGATACGACAACACT	CGGCTAGCACCATTTGAAATC	ACTG-CAGGGTCCGAGGTATT
miR-567	GTCGTATCGACTG-CAGGGTCCGAGGTATTCG-CAGTCGATACGACGTTCTG	CGGCAGTATGTTCTTCCAGGA	ACTG-CAGGGTCCGAGGTATT
miR-15b-3p	GTCGTATCGACTG-CAGGGTCCGAGGTATTCG-CAGTCGATACGACCCAAGT	CGGCCGAATCATTATTTGCT	ACTG-CAGGGTCCGAGGTATT
U6		CTCGCTTCGGCAGCACA	AACGCTTCAC-GAATTTGCGT
